# Predictive value of patent foramen ovale diameter for cryptogenic stroke and age-related differences

**DOI:** 10.3389/fcvm.2025.1647313

**Published:** 2025-08-21

**Authors:** Rui Wang, Miao Qiao, Guanghui Song, Wei Wang, Liuyin Yang, Zhenzhen Lin, Lingcui Meng

**Affiliations:** Department of Ultrasound Imaging, The Second Affiliated Hospital of Guangzhou University of Chinese Medicine, Guangzhou, Guangdong, China

**Keywords:** patent foramen ovale, cryptogenic stroke, right-to-left shunt, PFO diameter, age-related differences, stroke risk assessment

## Abstract

**Introduction:**

Patent foramen ovale (PFO) is associated with cryptogenic stroke (CS), whereas not all PFO carriers experience strokes. Current risk assessment tools like the Risk of Paradoxical Embolism (RoPE) scoring system and PFO-Associated Stroke Causal Likelihood (PASCAL) system have limitations, particularly in elderly populations. This study aims to explore risk factors for PFO-related CS and evaluate age-related differences between younger and elderly patients.

**Methods:**

This retrospective study included 344 patients with PFO, categorized into no stroke (NS), cryptogenic stroke (CS), and non-cryptogenic stroke (NCS) groups. Demographic, clinical, laboratory, and detailed PFO anatomical data were collected. Multivariate logistic regression and ROC analysis identified independent risk factors and optimal PFO diameter cut-off. Age subgroup analyses were performed.

**Results:**

17.2% of PFO patients were found to have CS. The mean PFO diameter was significantly larger in CS (2.54 ± 0.79 mm) compared to NS (1.70 ± 0.73 mm) and NCS (1.98 ± 1.10 mm; *P* < 0.05). Multivariate analysis confirmed PFO diameter as an independent CS risk factor (CS vs. NS: OR = 2.215, *P* = 0.001; CS vs. NCS: OR = 1.554, *P* = 0.028). ROC analysis demonstrated good predictive accuracy for CS (AUC = 0.773), with an optimal cut-off of 1.75 mm. Elevated white blood cell count (WBC), age ≥ 60 years, large right-to-left shunt (RLS), previous stroke/transient ischemic attack (TIA) and cortical infarction were associated with CS. Age subgroup analysis showed heterogeneity: in younger patients (<60 years), PFO diameter exhibited predictive capacity (AUC = 0.777, cut-off value = 1.75 mm) but lacked statistical significance in regression analysis (*P* > 0.05). Large RLS exhibited a risk factor (OR = 7.576, *P* = 0.099). Conversely, among elderly patients (≥60 years), PFO diameter remained a significant predictor (higher cut-off: 1.95 mm; AUC = 0.767), and smoking (OR = 5.26, *P* = 0.043) emerged an additional risk factor.

**Conclusion:**

CS was present in 17.2% of PFO patients. An enlarged diameter of PFO (optimal cut-off value: 1.75 mm in overall and younger; 1.95 mm in elderly) is a crucial anatomical risk factor. Elevated WBC, large RLS, previous stroke/TIA and cortical infarction are also correlated with CS. Age subgroup analysis revealed heterogeneity: PFO anatomy (diameter, RLS) is primary in younger patients, whereas in elderly patients (≥60 years), both PFO anatomy and systemic factors (smoking) should be considered.

## 1 Introduction

Stroke is the second leading cause of death worldwide, and 20%–30% of them are classified cryptogenic stroke (CS) (1). Patent foramen ovale (PFO), a common cardiac anatomical variant present in 20%–25% of the general adult population (2, 3), is detected in 40%–50% of young CS patients (4, 5). PFO is also implicated in multiple pathologies, including migraine with aura (6), decompression sickness (DCS) (7), and platypnea-orthodeoxia syndrome (8). The prevailing mechanistic hypotheses involve right-to-left shunt (RLS) and paradoxical embolism (9). However, not all PFO carriers develop strokes, with pathogenic risk modulated by anatomical features (e.g., PFO diameter, tunnel length, atrial septal aneurysm), RLS volume, and clinical factors such as inflammation or metabolic disturbances (10). Existing risk stratification tools, such as the Risk of Paradoxical Embolism (RoPE) scoring system (11) and the PFO-Associated Stroke Causal Likelihood (PASCAL) system (12), have limited applicability in elderly populations due to comorbidities and underweighted age contributions (RoPE assigns age only 1 or 0 points). Previous studies have shown that hypercoagulability (13), chronic inflammation, and metabolic issues can increase stroke risk by promoting thrombus formation, suggesting PFO's pathogenicity results from multiple factors.

Numerous studies have established the association between PFO and CS (14). However, the comprehensive clinical manifestations in PFO carriers ranging from asymptomatic status to various neurological symptoms (15), and their correlation with cerebrovascular abnormalities remain insufficiently characterized. Moreover, elderly patients were frequently excluded from CS studies due to confounding comorbidities (16), leaving PFO management in this population contentious. To address these critical gaps, this study aimed to: (a) establish the CS prevalence among consecutive PFO patients; (b) identify significant anatomical determinants (including PFO diameter, tunnel length, and RLS severity) and modifiable clinical risk factors; and (c) perform age subgroup analyses comparing younger (<60 years) and elderly (≥60 years) cohorts.

## 2 Methods

### 2.1 Study population

**Figure 1:**
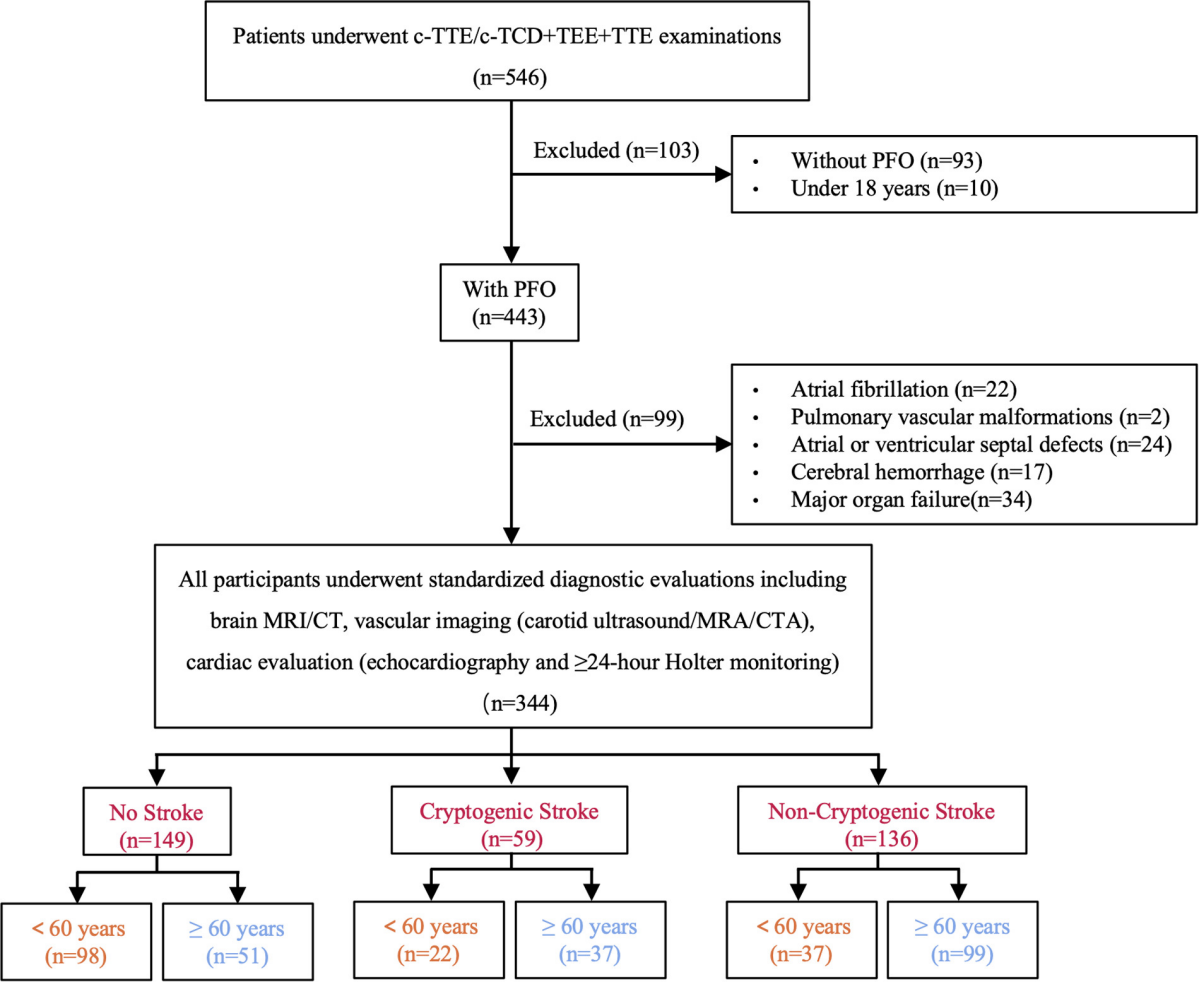
Flowchart of study population selection (TEE, transesophageal echocardiography; c-TCD, contrast-enhanced transcranial Doppler; c-TTE, contrast-enhanced transthoracic echocardiography; TTE, transthoracic echocardiography; MRI, magnetic resonance imaging; CT, computed tomography; CTA, computed tomography angiography; MRA, magnetic resonance angiography; PFO, patent foramen ovale).

This retrospective study was approved by the Institutional Review Board (IRB) of The Second Affiliated Hospital of Guangzhou University of Chinese Medicine (Ethics approval number: ZE2025-034-01). The requirement for written informed consent was waived by the IRB due to the retrospective nature of the study and use of anonymized data. All procedures followed the principles of the Declaration of Helsinki. TEE was performed for clinical evaluation of suspected PFO or CS in routine clinical practice, as part of standard diagnostic workup. Data were retrospectively collected for this study, with no additional examinations conducted specifically for this study. We enrolled 344 consecutive patients aged over 18 years admitted to our hospital between January 2021 and December 2024 who were diagnosed with PFO by transesophageal echocardiography (TEE), with intracardiac shunt confirmed by either contrast-enhanced transcranial doppler (c-TCD) or contrast-enhanced transthoracic echocardiography (c-TTE) (17). All participants underwent standardized diagnostic evaluations including brain MRI/CT, vascular imaging (carotid ultrasound/MRA/CTA), cardiac evaluation (echocardiography or 24 h Holter monitoring) to assess whether there was moderate or severe stenosis of the responsible artery, arrhythmias, or cardiac thrombi. All examinations were performed by experienced echocardiographers, and the results were assessed by another investigator, both of them were blinded to the patient's stroke status. Exclusion criteria included atrial fibrillation, pulmonary vascular malformations, atrial or ventricular septal defects, cerebral hemorrhage, and major organ failure (cardiac, hepatic, pulmonary, or renal) to ensure a homogeneous study population focused on PFO-related cerebrovascular outcomes (Figure 1).

### 2.2 General data

The baseline characteristics collected from medical records included: (1) demographic data: age, sex; (2) clinical history: hypertension, diabetes, smoking, previous stroke/transient ischemic attack (TIA), coronary heart disease; and (3) blood test data: white blood cell count (WBC), hemoglobin (Hb), red blood cell distribution width (RDW), platelet count (PLT), uric acid, creatinine, total cholesterol, triglyceride, low-density lipoprotein cholesterol (LDL-C), high-density lipoprotein cholesterol (HDL-C), glycated hemoglobin A1c (HbA1c), fibrinogen and D-dimer.

### 2.3 Anatomical features of PFO

The transesophageal echocardiogram examination was performed using the Philips EPIQ 7C color doppler ultrasound diagnostic apparatus. All measurements were calculated at rest and during a standardized Valsalva maneuver (sustained straining for over 5 s). PFO was defined as the separation between the septum primum and septum secundum with evidence of interatrial shunt. Quantitative measurements included: (1) PFO diameter (maximum separation between the septum primum and secundum on the left atrial side, in mm), measured 3 times at different cardiac cycles and averaged; (2) tunnel length (maximal visible overlap between the septum primum and secundum, in mm), measured 3 times at different cardiac cycles and averaged; and (3) left atrial appendage peak flow velocity (at end-systole, cm/s), measured 3 times at different cardiac cycles and averaged. The PFO tunnel length-to-diameter ratio (PFO tunnel length/diameter) was subsequently calculated for morphological assessment of PFO.

### 2.4 Right-to-left shunt (RLS)

The RLS was quantitatively evaluated by c-TCD with Delica EMS-9PB TCD detector or c-TTE by Philips EPIQ 7C color doppler ultrasound diagnostic apparatus. Assessments were performed at rest and during a standardized Valsalva maneuver (sustained straining for over 5 s and oral pressure reaching ≥40 mmHg).

The c-TCD classified RLS severity based on the number of microbubble signals detected in the middle cerebral artery (for unilateral middle cerebral artery monitoring; in cases of bilateral asymmetric shunting, the side with greater shunt volume was recorded). The grading evaluation criteria for RLS by c-TCD were as follows: Grade 0: No microbubbles were detected, indicating no shunt; Grade I: 1to10 microbubbles (1 to 20 bilaterally), indicating small RLS; Grade II: more than 10 microbubbles (more than 20 bilaterally), without curtain pattern, defining a moderate amount of RLS; Grade III: Curtain pattern of microbubble signals (individual bubbles indistinguishable), which was regarded as a large amount of RLS (18).

The grading evaluation criteria for RLS by c-TTE were as follows: Grading was based on the maximum number of microbubbles appearing in the left atrium on a single static frame image: Grade 0: No microbubbles were found in the left atrium, meaning no RLS; Grade I: The number of microbubbles per frame was less than 10, indicating a small amount of RLS; Grade II: There were 10 to 30 microbubbles per frame, indicating a moderate amount of RLS; Grade III: The number of microbubbles per frame was more than 30, or the left atrium was almost filled with microbubbles and the left atrium became opacified, which was considered as a large amount of RLS. Small RLS was defined as grade I/II RLS evaluated through c-TCD or c-TTE, while large RLS was defined as grade III RLS (19).

### 2.5 High-risk patent foramen ovale (PFO)

The anatomical features of high-risk PFO were defined according to the PASCAL Classification System (12): (1) the presence of a large RLS; and/or (2) the presence of atrial septal aneurysm (ASA), defined as the atrial septum protruding to one side with a depth exceeding the midline by 10 mm or the sum of bilateral swinging amplitudes being over 15 mm (20).

### 2.6 Definition of cryptogenic stroke (CS)

Cryptogenic stroke (CS) was defined according to the TOAST classification as ischemic stroke of undetermined etiology after exhaustive evaluation (21), including brain MRI/CT, vascular imaging (carotid ultrasound/MRA/CTA), cardiac evaluation (echocardiography and ≥24 h Holter monitoring), with exclusion of other potential causes (e.g., vasculitis or malignancy). Strokes with established etiologies (e.g., large-artery atherosclerosis, small artery disease) were classified as non-cryptogenic stroke (NCS), while cases without evidence of ischemic cerebrovascular disease were categorized as no stroke (NS). All diagnoses were confirmed by board-certified neurologists following the institutional stroke diagnostic protocol that adhered to current guidelines.

### 2.7 Statistical analyses

Statistical analyses were performed using SPSS 27.0 software. Descriptive statistics were used to summarize the baseline characteristics of the study population. Missing data for clinical and laboratory variables were minimal (≤5%), with no missing values observed for categorical variables. For continuous variables with missing values, imputation was performed based on their distribution characteristics: the mean of the corresponding group (NS, CS, or NCS) was used for normally distributed variables (assessed by the Shapiro–Wilk test or Q-Q plots), while the median of the respective group was applied to skewed variables.Continuous variables were presented as mean ± standard deviation (SD) if normally distributed and as median (interquartile range, IQR) otherwise. For continuous variables, one-way ANOVA was used if they followed a normal distribution with homogeneous variances; otherwise, the Kruskal–Wallis test was employed. Categorical variables were expressed as frequencies and percentages. The chi-square test was employed to compare groups and calculate odds ratio, and Fisher's exact test was adopted instead when sample size was small. Bonferroni correction was applied for multiple *post-hoc* comparisons.

Variables included in multivariate logistic regression models were selected based on a combination of prior knowledge (potential confounders identified from existing literature) and univariate analysis results (variables with *P* < 0.05 in univariate tests), ensuring both theoretical relevance and data-driven inclusion. Model validity was assessed via SPSS-generated outputs: acceptable goodness-of-fit was confirmed by a non-significant Hosmer-Lemeshow test (*P* > 0.05), and significant multicollinearity was ruled out with all variance inflation factor (VIF) values <5 derived from collinearity diagnostics. Then, a multivariate logistic regression analysis was carried out to analyze the relevant influencing factors, and a subgroup analysis was done according to age (<60 years vs. ≥60 years). To address potential type II errors, a *post-hoc* power analysis was conducted using PASS 15.0 (NCSS LLC). Power for logistic regression models was computed based on observed odds ratios with α = 0.05 (two-tailed). Power ≥80% was deemed adequate, 70%–79% marginal, and <70% inadequate. Subgroup analyses by age (<60 vs. ≥60 years) were explicitly evaluated for statistical power. The Receiver Operating Characteristic (ROC) curve was employed to evaluate predictive efficacy and determine cut-off values (determined by Youden's index), with *P* < 0.05 considered statistically significant.

## 3 Results

### 3.1 General information and risk factors

**Figure 2:**
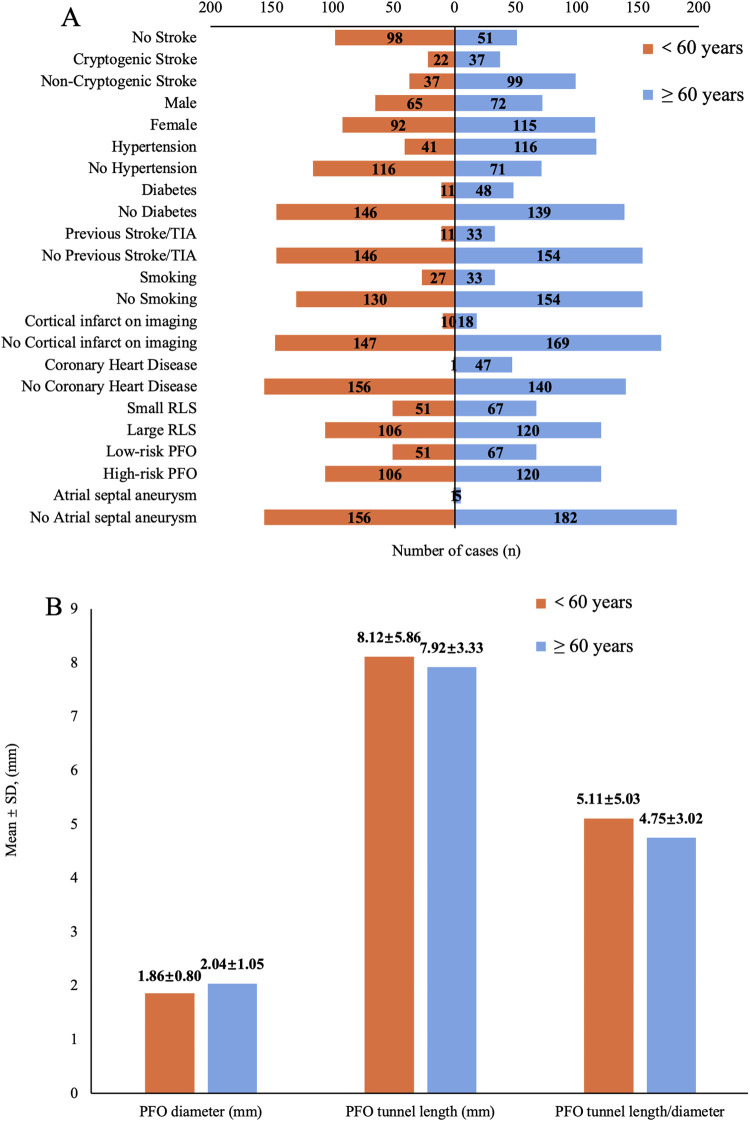
**(A)** Number distribution of demographic characteristics, comorbidities, and imaging parameters across age subgroups. (TIA, transient ischemic attack; RLS, right-to-left shunt; PFO, patent foramen ovale). **(B)** Mean ± SD of the patent foramen ovale (PFO) diameter, tunnel length and their ratio after subgroup analysis by age.

A total of 344 patients with PFO were enrolled in this study, including 149 cases in the no stroke group (NS, 43.3%), 59 cases in the cryptogenic stroke group (CS, 17.2%), and 136 cases in the non-cryptogenic stroke group (NCS, 39.5%), with CS having the lowest incidence. Baseline characteristics are summarized in Table 1. The NCS group was the oldest (64.13 ± 10.34 years), followed by CS (59.20 ± 12.67 years) and NS (52.77 ± 13.52 years). Age subgroup analysis revealed the lowest CS incidence in both younger (<60 years, 6.4%) and elderly (≥60 years, 10.8%) subgroups (Figure 2A), while NCS was most prevalent in the elderly (28.7%) and NS in the younger group (28.5%). Males predominated in NCS (18.6%), whereas females were more common in NS (30.8%). Hypertension, diabetes, smoking, Previous Stroke/TIA and coronary heart disease were most frequent in NCS (all *P* < 0.05), cortical infarction was significantly more frequent in CS compared to NCS (*P* < 0.001). PFO characteristics showed the largest PFO diameter in CS (2.54 ± 0.79 mm) and the lowest PFO tunnel length/diameter ratio (3.40 ± 1.60) (*P* < 0.05). Blood tests revealed higher WBC in NCS and CS vs. NS. Uric acid and creatinine were elevated in CS (*P* < 0.05), while total cholesterol peaked in NCS and triglycerides in NS. Triglyceride and HDL-C were higher in NS vs. NCS. HbA1c was significantly higher in CS (6.58 ± 2.20%) > NCS (6.12 ± 1.09%) > NS (5.71 ± 0.78%), *P* < 0.001.

**Table 1:** Baseline characteristics of patients with PFO, mean ± SD, median [IQR], *n* (%).

Variables	NS (*n* = 149)	CS (*n* = 59)	NCS (*n* = 136)	*P*-value
Age, years				
Age < 60, *n* (%)	98 (28.5)^a^	22 (6.4)^b^	37 (10.8)^b^	<0.001
Age ≥ 60, *n* (%)	51 (14.8)^a^	37 (10.8)^b^	99 (28.7)^b^	
Gender, sex				
Male, *n* (%)	43 (12.5)^a^	30 (8.7)^b^	64 (18.6)^b^	0.001
Female, *n* (%)	106 (30.8)^a^	29 (8.4)^b^	72 (20.9)^b^	
Hypertension				
Yes, *n* (%)	43 (12.5)^a^	28 (8.1)^b^	86 (25.0)^b^	<0.001
No, *n* (%)	106 (30.8)^a^	31 (9.0)^b^	50 (14.5)^b^	
Diabetes				
Yes, *n* (%)	9 (2.6)^a^	16 (4.7)^b^	34 (9.9)^b^	<0.001
No, *n* (%)	140 (40.7)^a^	43 (12.5)^b^	102 (29.7)^b^	
Previous Stroke/TIA				
Yes, *n* (%)	0 (0.0)^a^	17 (4.9)^b^	27 (7.8)^b^	<0.001
No, *n* (%)	149 (43.3)^a^	42 (12.2)^b^	109 (31.7)^b^	
Smoking				
Yes, *n* (%)	17 (4.9)^a^	16 (4.7)^b^	27 (7.8)^a^^,^^b^	0.017
No, *n* (%)	132 (38.4)^a^	43 (12.5)^b^	109 (31.7)^a^^,^^b^	
Cortical infarct on imaging				
Yes, *n* (%)	0 (0.0)^a^	16 (4.7)^b^	12 (3.5)^c^	<0.001
No, *n* (%)	149 (43.4)^a^	43 (12.5)^b^	124 (36.0)^c^	
Coronary heart disease				
Yes, *n* (%)	14 (4.1)^a^	6 (1.7)^a^^,^^b^	28 (8.1)^b^	0.016
No, *n* (%)	135 (39.2)^a^	53 (15.4)^a^^,^^b^	108 (31.4)^b^	
Atrial septal aneurysm				
Yes, *n* (%)	2 (0.6)^a^	0 (0)^a^	4 (1.2)^a^	0.313
No, *n* (%)	147 (42.7)^a^	59 (17.2)^a^	132 (38.3)^a^	
RLS				
Small RLS, *n* (%)	50 (14.5)^a^	8 (2.3)^b^	60 (17.4)^a^	<0.001
Large RLS, *n* (%)	99 (28.8)^a^	51 (14.8)^b^	76 (22.1)^a^	
Risk of PFO				
Low-risk PFO, *n* (%)	50 (14.5)^a^	8 (2.3)^b^	60 (17.4)^a^	<0.001
High-risk PFO, *n* (%)	99 (28.8)^a^	51 (14.8)^b^	76 (22.1)^a^	
Age, (years)	52.77 ± 13.52^a^	59.20 ± 12.67^b^	64.13 ± 10.34^c^	<0.001
PFO diameter, (mm)	1.70 ± 0.73^a^	2.54 ± 0.79^b^	1.98 ± 1.10^c^	<0.001
PFO tunnel length, (mm)	7.74 ± 4.27^a^	8.05 ± 3.09^a^	7.85 ± 3.71^a^	0.847
PFO tunnel length/diameter	5.16 ± 3.22^a^	3.40 ± 1.60^b^	4.92 ± 3.16^a^^,^^b^	0.001
LAA PSV, (cm/s)	71.86 ± 18.39^a^	71.12 ± 22.60^a^	72.40 ± 19.58^a^	0.917
WBC, (×10⁹/L)	6.46 ± 1.77^a^	7.02 ± 1.97^a^^,^^b^	7.06 ± 2.13^b^	0.025
Hemoglobin, (g/L)	130.42 ± 18.27^a^	132.78 ± 15.53^a^	131.65 ± 14.64^a^	0.616
RDW, (%)	13.02 ± 1.00^a^	13.00 ± 1.09^a^	13.15 ± 1.00^a^	0.480
PLT, (×10⁹/L)	246.47 ± 68.40^a^	233.14 ± 51.72^a^	234.97 ± 59.86^a^	0.206
Uric Acid, (μmol/L)	317.95 ± 88.57^a^	355.37 ± 100.98^b^	334.63 ± 94.65^a^^,^^b^	0.043
Creatinine, (μmol/L)	63.00 [15.00]^a^	76.50 [31.3]^b^	71.50 [23.3]^c^	<0.001
Total Cholesterol, (mmol/L)	3.23 ± 1.91^a^	3.62 ± 1.68a^a^^,^^b^	3.99 ± 1.66^b^	0.002
Triglyceride, (mmol/L)	1.93 [1.86]^a^	1.68 [1.30]^a^^,^^b^	1.37 [1.03]^b^	0.006
HDL-C, (mmol/L)	2.96 ± 2.02^a^	1.84 ± 1.47^b^	1.82 ± 1.50^c^	<0.001
LDL-C, (mmol/L)	2.30 [1.86]^a^	2.35 [1.52]^a^	2.61 [1.37]^a^	0.093
HbA1c, (%)	5.71 ± 0.78^a^	6.58 ± 2.20^b^	6.12 ± 1.09^c^	<0.001
D-dimer, (mg/L)	0.62 [0.14]^a^	0.84 [0.26]^a^	0.63 [0.32]^a^	0.453
Fibrinogen, (g/L)	2.98 ± 0.69^a^	3.13 ± 1.00^a^	2.90 ± 0.91^a^	0.222

^a,b,c^ Different superscript letters (a, b, c) indicate significant differences between groups (*P* < 0.05, Bonferroni-adjusted).

TIA, transient ischemic attack; ASA, atrial septal aneurysm; RLS, right-to-left shunt; PFO, patent foramen ovale; LAA, left atrial appendage; PSV, peak systolic velocity; WBC, white blood cell count; RDW, red blood cell distribution width; PLT, platelet count; LDL-C, low-density lipoprotein cholesterol; HDL-C, high-density lipoprotein cholesterol; HbA1c, glycated hemoglobin A1c; NS, no stroke; CS, cryptogenic stroke; NCS, non-cryptogenic stroke.

### 3.2 Influencing factors of CS related to PFO in general

Multivariate logistic regression analysis (Table 2) results showed that increased PFO diameter is an independent risk factor for CS (CS vs. NS: OR = 2.215, *P* = 0.001; CS vs. NCS: OR = 1.554, *P* = 0.028). The presence of cortical infarction was a strong independent risk factor for CS (CS vs. NCS: OR = 8.333, 95%CI = 2.604–26.32, *P* < 0.001). Nonetheless, due to the small number of cortical infarction positive cases, the reliability of this result needs to be verified with larger samples. In the comparison between CS and NS, elevated WBC (CS vs. NS: OR = 1.258, *P* = 0.043) and age ≥60 years (CS vs. NS: OR = 8.264, *P* = 0.004) increased the risk of CS. In the comparison between CS and NCS, large RLS (CS vs. NCS: OR = 4.902, *P* = 0.004) and previous stroke/TIA (OR = 2.793, *P* = 0.045) were independently associated with an increased risk of CS. These findings suggested that elevated WBC, age ≥60, large RLS, and previous stroke/TIA are risk factors for CS. Other variables (e.g., HbA1c, diabetes) showed no significant associations.

**Table 2:** Multivariate logistic regression analysis of the influencing factors for cryptogenic stroke related to patent foramen ovale (PFO) in general.

Variables	CS vs. NS				CS vs. NCS			
	B	*P-*value	OR	95%CI	B	*P-*value	OR	95%CI
PFO diameter	0.795	0.001	2.215	1.375–3.568	0.441	0.028	1.554	1.050–2.299
WBC	0.229	0.043	1.258	1.007–1.571	0.052	0.588	1.053	0.873–1.270
HbA1c	0.444	0.198	1.558	0.793–3.063	0.404	0.129	1.498	0.889–2.522
Age								
Age < 60	−2.110	0.004	0.121	0.028–0.518	−0.709	0.287	0.492	0.133–1.816
Age ≥ 60			8.264	1.931–35.71			2.033	0.551–7.519
Diabetes								
No	−0.231	0.806	0.794	0.126–5.021	0.799	0.279	2.223	0.523–9.444
Yes			1.259	0.199–7.937			0.450	0.106–1.912
Previous Stroke/TIA								
No	−19.091	0.989	–^a^	–^a^	−1.026	0.045	0.358	0.131–0.979
Yes							2.793	1.021–7.634
Cortical infarct on imaging								
No	−20.435	<0.001	–^a^	–^a^	−2.118	<0.001	0.120	0.038–0.384
Yes							8.333	2.604–26.32
Small RLS	−0.577	0.346	0.561	0.169–1.865	−1.590	0.004	0.204	0.070–0.595
Large RLS							4.902	1.681–14.29

^a^ Odds ratio not calculable due to complete separation.

NS, no stroke; CS, cryptogenic stroke; NCS, non-cryptogenic stroke; TIA, transient ischemic attack; RLS, right-to-left shunt; PFO, patent foramen ovale; WBC, white blood cell count; HbA1c, glycated hemoglobin A1c.

### 3.3 Influencing factors of CS related to PFO in subgroup analysis by age

In the age subgroup analysis, significant differences emerged between younger (<60 years) and elderly (≥60 years) patients. Among younger individuals, the odds ratio (OR) values of PFO diameter (CS vs. NS: OR = 2.129, *P* = 0.120; CS vs. NCS: OR = 1.591, *P* = 0.329) and WBC (CS vs. NS: OR = 1.403, *P* = 0.185; CS vs. NCS: OR = 1.229, *P* = 0.373) increased when comparing CS and NS, yet neither reached statistical significance(*P* > 0.05). Large RLS exhibited a potential risk trend when comparing CS and NCS (OR = 7.576, *P* = 0.099). Other variables (e.g., HbA1c, diabetes) in this age group also showed no significant associations (Table 3A). However, in elderly patients (Table 3B), PFO diameter remained a strong independent predictor (CS vs. NS: OR = 2.446, *P* = 0.009; CS vs. NCS: OR = 1.737, *P* = 0.037), and also larger when compared to younger (2.04 ± 1.05 mm vs. 1.86 ± 0.80 mm) (Figure 2B). Notably, the presence of cortical infarction was a strong independent risk factor for CS (CS vs. NCS: OR = 30.303, *P* < 0.001), still needs to be verified with larger samples. Furthermore, smoking status emerged as a significant risk factor when comparing CS to NCS (OR = 5.263, *P* = 0.043), suggesting the history of smoking increases CS risk in elderly. No other variables showed significant associations in the elderly group.

**Table 3A:** Multivariate logistic regression analysis of the influencing factors for cryptogenic stroke related to PFO in people aged < 60 years.

Variables	CS vs. NS				CS vs. NCS			
	B	*P* value	OR	95% CI	B	*P* value	OR	95% CI
PFO diameter	0.755	0.120	2.129	0.821–5.517	0.464	0.329	1.591	0.626–4.040
WBC	0.339	0.185	1.403	0.851–2.316	0.207	0.373	1.229	0.780–1.937
HbA1c	1.597	0.102	4.938	0.729–33.46	1.019	0.282	2.770	0.443–17.71
Diabetes								
No	4.019	0.232	55.623	0.08–40,361	1.896	0.541	6.660	0.015–2,928
Yes			0.018	0.000–12.5			0.150	0.00–66.667
Previous Stroke/TIA								
No	−19.584	0.986	–^a^	–^a^	−1.495	0.296	0.244	0.014–3.708
Yes							4.098	0.27–71.429
Cortical infarct on imaging								
No	−16.436	0.982	–^a^	–^a^	0.471	0.790	1.601	0.050–51.07
Yes							0.625	0.020–20.00
Small RLS	−0.723	0.566	0.485	0.041–5.753	−2.028	0.099	0.132	0.012–1.463
Large RLS			2.062	0.174–24.39			7.576	0.684–83.33

^a^ Odds ratio not calculable due to complete separation.

NS, no stroke; CS, cryptogenic stroke; NCS, non-cryptogenic stroke; TIA, transient ischemic attack; RLS, right-to-left shunt; PFO, patent foramen ovale; WBC, white blood cell count; HbA1c, glycated hemoglobin A1c.

**Table 3B:** Multivariate logistic regression analysis of the influencing factors for cryptogenic stroke related to PFO in people aged ≥ 60 years.

Variables	CS vs. NS				CS vs. NCS			
	B	*P* value	OR	95% CI	B	*P* value	OR	95% CI
PFO diameter	0.895	0.009	2.446	1.251–4.783	0.552	0.037	1.737	1.033–2.922
WBC	0.143	0.319	1.154	0.871–1.529	0.015	0.903	1.015	0.802–1.284
HbA1c	0.085	0.865	1.089	0.409–2.898	0.260	0.427	1.297	0.683–2.464
Diabetes								
No	−1.202	0.319	0.301	0.028–3.200	0.685	0.428	1.985	0.364–10.81
Yes			3.322	0.313–35.71			0.504	0.093–2.747
Previous Stroke/TIA								
No	−16.711	0.988	–^a^	–^a^	−0.852	0.176	0.427	0.124–1.464
Yes							2.342	0.683–8.065
Cortical infarct on imaging								
No	−20.125	<0.001	–^a^	–^a^	−3.412	<0.001	0.033	0.006–0.174
Yes							30.303	5.747–166.7
Smoking								
No	−1.312	0.175	0.296	0.040–1.795	−1.661	0.043	0.190	0.038–0.952
Yes			3.378	0.557–25.00			5.263	1.05–26.316
Small RLS	−0.205	0.801	0.814	0.165–4.009	−1.008	0.143	0.365	0.095–1.406
Large RLS			1.229	0.249–6.061			1.575	0.711–10.53

^a^ Odds ratio not calculable due to complete separation.

NS, no stroke; CS, cryptogenic stroke; NCS, non-cryptogenic stroke; TIA, transient ischemic attack; RLS, right-to-left shunt; PFO, patent foramen ovale; WBC, white blood cell count; HbA1c, glycated hemoglobin A1c.

### 3.4 Post-hoc power analysis

A *post-hoc* power analysis showed that the association between PFO diameter and cryptogenic stroke (CS) had sufficient statistical power in the overall population, with power exceeding 80% in comparisons of CS vs. NS (82.3%) and CS vs. NCS (81.5%). In the young subgroup (<60 years), however, the statistical power was inadequate, being 61.8% for CS vs. NS and 58.2% for CS vs. NCS. For the elderly subgroup (≥60 years), the power was marginal, at 76.2% for CS vs. NS and 71.0% for CS vs. NCS. For the younger subgroup, the power was <70%, which may explain why PFO diameter did not reach statistical significance in the regression analysis for this subgroup (despite the OR suggesting a potential association); this indicates that the smaller sample size in the younger patient subgroup may have limited the ability to detect effects, requiring cautious interpretation. In the elderly subgroup, the power was from 70%–80% (close to 80%), supporting the credibility of PFO diameter as a significant predictor in this subgroup. Detailed parameters are provided in Supplementary Table S1.

### 3.5 ROC analysis of PFO diameter for cryptogenic stroke prediction

**Figure 3:**
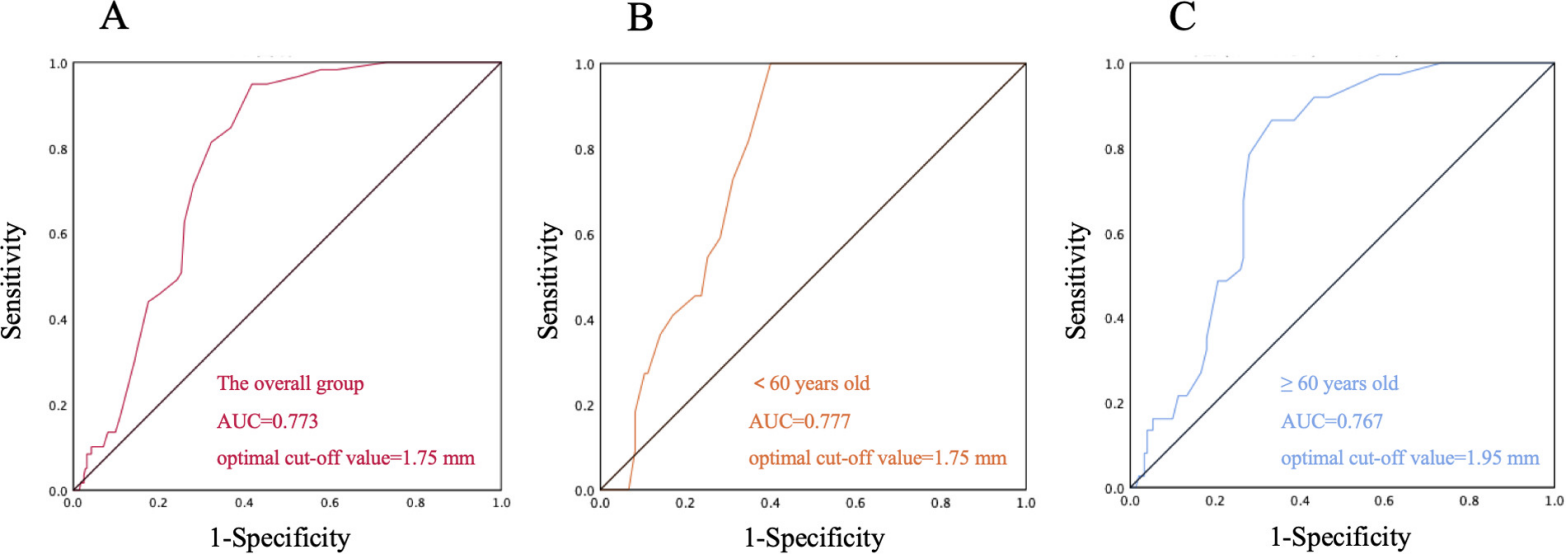
Receiver Operating Characteristic (ROC) curve of patent foramen ovale (PFO) diameter for cryptogenic stroke prediction. **(A)** ROC curve of the overall group; **(B)** ROC curve of the subgroup aged < 60 years; **(C)** ROC curve of the subgroup aged ≥ 60 years.

ROC curve analysis demonstrated that the PFO diameter exhibited a good predictive accuracy for CS in the overall population (AUC = 0.773, 95% CI: 0.722–0.823) (Figure 3A), and the optimal cut-off value was determined to be 1.75 mm. This association remained significant in age subgroup analysis. Notably, the elderly group (≥60 years) exhibited a higher optimal cut-off (1.95 mm, AUC = 0.767, 95% CI: 0.679–0.836) compared to the younger group (<60 years; 1.75 mm, AUC = 0.777, 95% CI: 0.704–0.851) (Figures 3B,C).

## 4 Discussion

This study investigated the association between PFO and stroke by analyzing 344 patients diagnosed with PFO via transesophageal echocardiography. The patients were categorized into no stroke group (NS, 43.3%), cryptogenic stroke group (CS, 17.2%), and non-cryptogenic stroke group (NCS, 39.5%). The NCS group was oldest (mean 64.13 years), followed by CS (59.20 years) and NS (52.77 years), reflecting an age-related risk gradient. Despite this, the CS group accounted for the smallest proportion (6.4%) in both age subgroups (<60 and ≥60 years), indicating that most strokes in PFO patients are linked to traditional risk factors rather than paradoxical embolism alone, suggesting PFO may often be incidental finding rather than a direct stroke cause (22). Additionally, the NCS group had a higher proportion of males (18.6%), whereas females predominated in the NS group (30.8%), consistent with previous findings that older males are more susceptible to atherosclerosis-related stroke while females with PFO may have relatively lower stroke risk (23). In terms of risk factors, hypertension and diabetes were most prevalent in the NCS group, reinforcing the predominant role of traditional vascular risk factors in non-cryptogenic stroke (24). However, the CS group exhibited significantly higher HbA1c levels (6.58 ± 2.20% in CS vs. 5.71 ± 0.78% in NS) (25), along with elevated uric acid (26), creatinine, and WBC. These metabolic disturbances suggest poor glycemic control, impaired renal function, and systemic inflammation (27) may contribute to CS. Anatomically, PFO diameter was significantly larger in the CS group (2.54 mm vs. 1.70 mm in NS), supporting the hypothesis that larger PFO size increases paradoxical embolism risk. Collectively, while PFO-related strokes are less common than traditional etiologies, the convergence of metabolic dysfunction, pro-inflammatory activity, and specific PFO morphology appears to synergistically increases CS risk (28).

Multivariate logistic regression revealed that the PFO diameter (2.54 ± 0.79 mm) in the CS group was significantly larger than that in the NCS and NS groups (*P* < 0.05), indicating it as an independent risk factor for PFO-related CS (29, 30). Consistent with this, ROC curve analysis demonstrated good predictive accuracy of PFO diameter for CS in the overall cohort (AUC = 0.773), with an optimal cut-off value of 1.75 mm, suggesting that PFO diameter >1.75 mm may serve as a clinically relevant risk factor for stroke. Moreover, the ratio of PFO tunnel length to diameter (3.40 ± 1.60) was smaller in the CS group, implying that a short and wide PFO structure might predispose to paradoxical embolism, although this association did not reach statistical significance (*P* > 0.05). Notably, the NS group comprised the highest proportions of patients aged <60 years, those with large RLS, and high-risk PFO, likely attributable to younger age, enhanced Valsalva efficacy, and fewer vascular risk factors (31). The analysis also revealed that previous stroke/TIA was significantly associated with CS, potentially mediated by vascular remodeling and hemodynamic alterations that increase recurrence susceptibility, or undetermined underlying pathologies (32). Cortical infarction emerged as another significant CS predictor (*P* < 0.001), aligning with previous studies indicating that infarctions caused by PFO are more likely to occur in the subcortical regions of the superficial areas of the brain (33). However, the limited number of cortical infarction-positive cases necessitates validation in larger cohorts to confirm the reliability of this association. Elevated white blood cell count in CS (*P* < 0.05 vs. NS, OR = 1.258), further implicated pro-thrombotic inflammatory mechanisms. Collectively, these findings underscore the multifactorial nature of CS risk, necessitating integrated assessment of anatomical, metabolic, and inflammatory markers beyond PFO characteristics alone.

Age subgroup analysis (<60 years) revealed no statistically significant predictors of CS in multivariate logistic regression. However, the odds ratio (OR) of the PFO diameter and WBC count increased without attaining statistical significance (*P* > 0.05), this pattern might be attributed to the limited power from small sample size or residual confounding. ROC curve analysis demonstrated good predictive accuracy of PFO diameter for CS in this subgroup (AUC = 0.777), with an identical optimal cut-off (1.75 mm) to the overall cohort, suggesting its potential clinical relevance despite non-significance in regression. Notably, large RLS exhibited a potential Pathogenicity (OR = 7.576, *P* = 0.099), which aligns with prior evidence (34) and implies that large RLS may be mechanistically more critical for PFO-related stroke in younger patients. Traditional vascular risk factors didn't have a significant impact on the risk of CS in this group, which implies that the stroke mechanism of younger CS patients might rely more on PFO's anatomical features or unevaluated hypercoagulable states (35, 36). Some related indicators like situ microthrombus weren't detected in this study and could be explored further (37).

**Figure 4:**
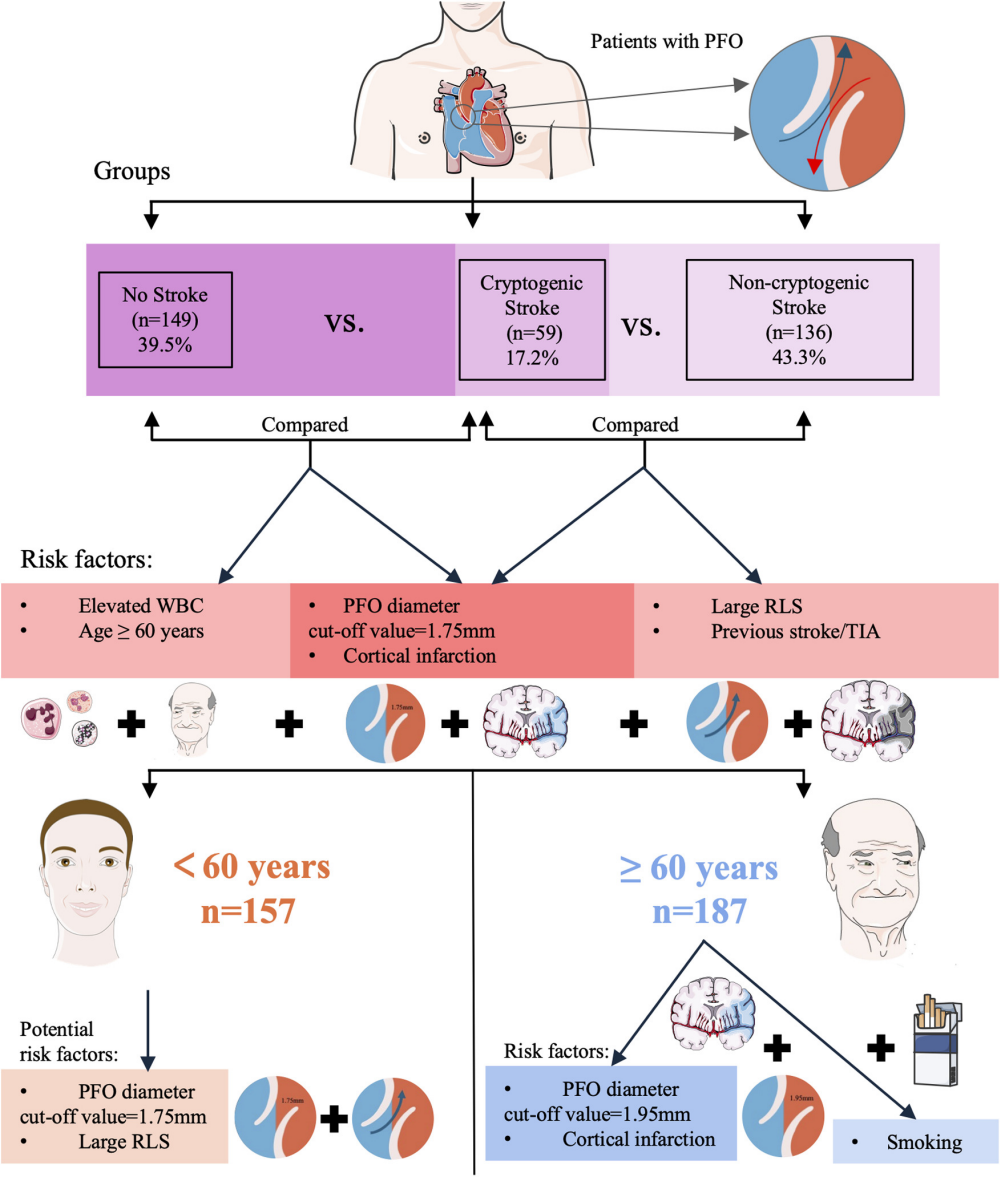
Visual summary of patient groups, risk factors for cryptogenic stroke, and age subgroup analysis. (TIA, Transient ischemic attack; RLS, right-to-left shunt; PFO, patent foramen ovale; WBC, white blood cell count).

Among elderly patients (≥60 years), PFO diameter retained stronger predictive value (OR = 2.446 vs. 2.129 in young) with a larger mean diameter (2.04 ± 1.05 mm vs. 1.86 ± 0.80 mm in young) and higher optimal threshold (1.95 mm vs. 1.75 mm in young). This likely reflected age-related hemodynamic changes. Mechanistically, a larger PFO diameter lowers the threshold for paradoxical embolism, enabling venous thrombi to more readily cross the interatrial septum into the systemic circulation and increasing the risk of cerebral artery occlusion (38). This aligns with our finding that PFO diameter in the CS group was significantly larger than in NS groups. Consistent with prior evidence, PFO prevalence decreases while diameter increases with aging (39), which further lowers the threshold for paradoxical embolism. Concurrently, elderly patients often exhibit age-related hemodynamic changes (e.g., elevated right atrial pressure due to pulmonary hypertension or diastolic dysfunction), which further augment RLS shunt volume through a larger PFO, thereby amplifying embolic risk (40, 41). Superimposed on these anatomical and hemodynamic factors, the elderly population commonly presents with endothelial dysfunction (42), chronic inflammatory states (partially reflected by elevated WBC counts in our CS cohort), and procoagulant tendencies—all of which inherently increase thrombus formation risk—while a larger PFO provides a more efficient conduit for such thrombi. Additionally, ASA is more prevalent in elderly patients, may create a local prothrombotic microenvironment via blood stasis, synergizing with enlarged PFO diameter to further exacerbate stroke risk (43). Smoking was an additional risk factor in the elderly (OR = 5.263, *P* = 0.043), reinforcing its established role in stroke via pro-inflammatory endothelial injury (44). Collectively, these results underscore the imperative for comprehensive PFO anatomical assessment (diameter, ASA) and vascular risk profiling in elderly cryptogenic stroke management (45). A visual summary of these findings is provided in Figure 4.

The *post-hoc* power analysis offers important perspectives on the reliability of the associations between PFO diameter and CS across different populations. In the overall cohort, sufficient statistical power (>80%) in comparisons of CS vs. NS (82.3%) and CS vs. NCS (81.5%) reinforces the validity of PFO diameter as an independent risk factor for CS, consistent with primary findings. The inadequate power in the younger subgroup (<60 years; 61.8% for CS vs. NS and 58.2% for CS vs. NCS) helps account for why PFO diameter did not reach statistical significance in multivariate regression for this group, despite ROC analysis suggesting potential predictive capacity (AUC = 0.777, cut-off = 1.75 mm), highlighting the need for caution in interpreting results here, even as large RLS showed a trend toward significance (OR = 7.576, *P* = 0.099). Conversely, the marginal yet higher power in the elderly subgroup (≥60 years; 76.2% for CS vs. NS and 71.0% for CS vs. NCS) supports the significance of PFO diameter as a predictor in this population, where a higher cut-off (1.95 mm) was identified, aligning with the observation that both PFO anatomical features and systemic factors like smoking contribute to CS risk in older patients. Overall, the power analysis underscores the importance of considering sample size and age-related heterogeneity when evaluating the clinical relevance of PFO diameter in CS risk assessment.

The identified cutoffs (1.75 mm overall/younger; 1.95 mm elderly) may guide risk stratification: Patients exceeding these values should be prioritized for closer monitoring. In younger patients, a diameter exceeding 1.75 mm (indicating large RLS) could inform closure decisions. In the elderly, management should integrate PFO assessment (diameter >1.95 mm) with control of modifiable risk factors (e.g., smoking cessation). Elevated WBC or cortical infarction should prompt comprehensive thrombophilia screening; such patients could benefit from closer monitoring or consideration of PFO closure, thus complementing existing tools like the RoPE scoring system (11) or PASCAL system (12). However, these cutoffs are derived from exploratory ROC analysis and require validation in prospective independent cohorts before clinical application.

Our study demonstrated an association between PFO diameter and CS; however, the cross-sectional design precludes causal inference. Residual confounding due to unmeasured factors—such as subclinical atrial fibrillation (AF) despite standard cardiac monitoring, undiagnosed thrombophilia, or undocumented prior venous thromboembolism (VTE)—may introduce bias. Future studies should incorporate long-term cardiac monitoring and thrombophilia screening. Although pathophysiological mechanisms (e.g., paradoxical embolism) are likely generalizable, ethnic variations in PFO prevalence necessitate validation in multinational cohorts.

This study had several limitations. First, as a tertiary referral center, our cohort may overrepresent cases with complex PFO anatomy or severe strokes, potentially limiting generalizability to community-based populations. Additionally, the retrospective design may introduce selection bias, particularly because patients with small RLS did not routinely undergo confirmatory TEE. Second, smaller subgroups—such as younger patients and those with CS—limited statistical power for nuanced analyses. Third, residual confounding from unmeasured factors —such as subclinical atrial fibrillation (46), undiagnosed thrombophilia, or undocumented VTE—may have influenced the observed associations, as these factors could independently contribute to stroke risk or interact with PFO features. Fourth, we did not evaluate non-stroke PFO-related conditions (e.g., migraines), nor were these systematically categorized. Future multicenter studies with broader enrollment are needed to clarify the role of PFOs in diverse clinical presentations and patient populations.

## 5 Conclusion

Cryptogenic stroke was present in 17.2% of PFO patients. Multivariate analysis confirmed PFO diameter as an independent CS risk factor. ROC analysis showed good predictive accuracy for CS, with optimal predictive cut-off values of 1.75 mm (overall cohort and patients <60 years) and 1.95 mm (patients ≥60 years). Additionally, inflammation (elevated WBC), RLS, previous stroke/TIA, and cortical infarction were associated with CS. Age subgroup analysis revealed heterogeneity: in younger patients (<60 years), PFO anatomy (diameter, RLS) was the primary focus, whereas in elderly patients (≥60 years), both PFO anatomy (diameter, ASA) and systemic factors (smoking) should be considered. Personalized evaluation based on age is essential. These findings advocate for personalized risk stratification in PFO-associated stroke, incorporating age-specific anatomical features, vascular risk burden, and inflammatory biomarkers.

## Data Availability

The original contributions presented in the study are included in the article/Supplementary Material, further inquiries can be directed to the corresponding author.
