# Supplementary material for: Predictive value of patent foramen ovale diameter for cryptogenic stroke and age-related differences
Source: Front Cardiovasc Med. 2025 Aug 21;12:1647313. doi: 10.3389/fcvm.2025.1647313 (PMC12408557; doi:10.3389/fcvm.2025.1647313)
Supplement: Supplementary file 1 [file Table1.pdf]

frontiers

Supplementary Material

TABLE S1 Statistical Power of PFO Diameter and CS Association in Overall and Age-Specific Subgroups

Comparison Group	Sample Size (CS/Control)	Observed OR	Statistical Power
Overall			
CS vs NS	208 (59CS/149NS)	2.215	82.3%
CS vs NCS	195 (59CS/136NCS)	1.554	81.5%
Young subgroup (<60y)			
CS vs NS	120 (22CS/98NS)	2.129	61.8%
CS vs NCS	59 (22CS/37NS)	1.591	58.2%
Elderly subgroup (≥60y)			
CS vs NS	88 (37CS/51NS)	2.446	76.2%
CS vs NCS	136 (37CS/99NS)	1.737	71.0%

Abbreviations: PFO: Patent foramen ovale, NS: No Stroke, CS: Cryptogenic Stroke, NCS: Non-Cryptogenic Stroke, OR: Odds Ratio. Covariate R^2=0.2, α=0.05 two-tailed, Power ≥80% was deemed adequate, 70–79% marginal, and <70% inadequate.
